# A Case of Fistula in Ano, Accompanied with the Lodgement of a Plumb Stone in the Rectum

**Published:** 1800-03

**Authors:** Thomas Whately

**Affiliations:** Member of the Corporation of Surgeons of London


### *To the Editors of the Medical and Physical Journal.*

GENTLEMEN,

IF you deem the following Case of Fistula in Ano, worthy of a place in your valuable publication, it is at your service.

I am,

GENTLEMEN,

Your most obedient humble servant,

THO. WHATELY.

*Bedford-Row, Feb.* 4, 1800.

---

### *A Case of Fistula in Ano, accompanied with the Lodgement of a Plumb Stone in the Rectum; by* THOMAS WHATELY, Member of the Corporation of Surgeons of London.

Mr. Chipperfield, at the Bell, Great Carter-lane, Doctors-Commons, aged 45, applied to me in September, 1799, for the cure of a fistula in ano, of more than ten years standing. He had consulted a surgeon on his case, at a very early period of of the diſeaſe, but was told, that he had not that complaint. The fiſtula continued nearly ſeven years in the ſame ſtate as at its firſt appearance. About the expiration of this time, he was ſeized with a violent diarrhœa, attended with conſiderable pain in the bowels, particularly in the rectum, and about the anus, where he very frequently felt a pricking and ſhooting. He was not able, after an evacuation, to cloſe the ſphincter ani without great pain, which generally continued for three or four minutes: the ſymptoms continued with ſuch violence only two or three days; but from this time till he applied to me, (a period of more than three years,) he has been afflicted with a diarrhœa, and has generally had about four or five ſtools every day, and for the moſt part one every night. At each motion he was obliged to obey the moment the call came upon him. During this period he felt occaſionally ſome pain in the abdomen, and a pricking about the rectum. At intervals his ſtools would be ſomewhat ſolid; and he remembers to have frequently obſerved a diviſion, or mark, on one ſide of the fæces, as if ſomething had cut them in their paſſage, which he compared to a bullet, ſhot from a rifle-barrelled gun. About three or four days previous to his application to me, he was again ſeized with a violent diarrhœa, attended nearly with the ſame ſymptoms as at the prior attack. It was very ſevere for two days, and then almoſt entirely ceaſed; but the pain in the lower part of the abdomen, and about the anus, continued without much intermiſſion. In the day-time, it extended round his loins, and was often ſo violent on riſing from a chair, as to prevent his walking erect. In the night he was very reſtleſs, and for the moſt part deprived of ſleep.

Conceiving that he was not in a fit ſtate to undergo an operation, I adviſed him to take ſome doſes of Caſtor oil; and after theſe, ſome opiates. By theſe means his complaints were leſſened; but they were by no means ſo much abated at the end of a week as I expected. From a ſuſpicion that there might be ſomething about the rectum, diſtinct from the fiſtula, from which his complaints aroſe, I begged him to let me examine the gut with my finger. To my great ſurprize, I ſoon felt a ſtone within the rectum, which I afterwards extracted by a pair of forceps. From this time all his complaints, except the fiſtula, entirely ceaſed, and he ſoon recovered a perfect ſtate of health. On examining the ſtone, I found it eat away on each ſide to a conſiderable depth; an edge nearly as ſharp as that of a knife being left. By this circumſtance, no doubt, all his complaints had been greatly aggravated.

At the end of ten days from the extraction of the ſtone, I cut him for the fiſtula. On introducing my finger into the rectum for that purpose, I found the sphincter narrower than usual, which, probably, arose from the long continuance of the fistula. I could plainly feel within the gut, a kind of nidus, formed by a large fold of its internal coat; in this the stone had lain. The fistulous cavity communicated with the rectum higher up than it commonly does. From the opening in this part, the cavity of the rectum, and that of the fistula, were, by the operation, laid into one; after which the patient obtained a perfect cure in about a month.

From the history of this case, it appears very clearly, that the plumb stone had continued in the lowest part of the rectum upwards of three years. What prevented its discharge from this situation at any of the evacuations of the gut, was, probably, the contraction of the sphincter, occasioned by the long continuance of the fistula, previous to his swallowing the stone. These conclusions are grounded on the length of time the diarrhoea continued, the eroded state of the plumb stone, the ceasing of the diarrhoea from the time of the extraction of the stone, and the patient's having a better appetite, and a better state of health, after the extraction of the stone, than he had experienced for the three preceding years.

